# First record of a proseriate flatworm predating on a rhabdocoel (Platyhelminthes: Proseriata and Rhabdocoela)

**DOI:** 10.3897/BDJ.12.e116039

**Published:** 2024-05-13

**Authors:** Claudia Sanjuan Hernández, Marco Curini-Galletti, Marlies Monnens, Tom Artois, Yander L. Diez

**Affiliations:** 1 Universidad de Oriente, Biology & Geography Department, Ave. Patricio Lumumba s/n, CP 90500, Santiago de Cuba, Cuba Universidad de Oriente, Biology & Geography Department, Ave. Patricio Lumumba s/n, CP 90500 Santiago de Cuba Cuba; 2 Hasselt University, Centre for Environmental Sciences, Research Group Zoology: Biodiversity and Toxicology, Universitaire Campus Gebouw D, B-3590, Diepenbeek, Belgium Hasselt University, Centre for Environmental Sciences, Research Group Zoology: Biodiversity and Toxicology, Universitaire Campus Gebouw D, B-3590 Diepenbeek Belgium; 3 Università di Sassari, Dipartimento di Medicina Veterinaria, Sardinia, Italy Università di Sassari, Dipartimento di Medicina Veterinaria Sardinia Italy; 4 4Royal Belgian Institute of Natural Sciences, OD Taxonomy and Phylogeny, Vautierstraat 29, B-1000, Brussels, Belgium 4Royal Belgian Institute of Natural Sciences, OD Taxonomy and Phylogeny, Vautierstraat 29, B-1000 Brussels Belgium; 5 2Hasselt University, Centre for Environmental Sciences, Research Group Zoology: Biodiversity and Toxicology, Universitaire Campus Gebouw D, B-3590, Diepenbeek, Belgium 2Hasselt University, Centre for Environmental Sciences, Research Group Zoology: Biodiversity and Toxicology, Universitaire Campus Gebouw D, B-3590 Diepenbeek Belgium; 6 Museum of Nature Hamburg, Leibniz Institute for the Analyses of the Biodiversity Change, Hamburg, Germany Museum of Nature Hamburg, Leibniz Institute for the Analyses of the Biodiversity Change Hamburg Germany; 7 2Hasselt University, Centre for Environmental Sciences, Research Group Zoology: Biodiversity and Toxicology, Universitaire Campus Gebouw D, B-3590, Hasselt, Belgium 2Hasselt University, Centre for Environmental Sciences, Research Group Zoology: Biodiversity and Toxicology, Universitaire Campus Gebouw D, B-3590 Hasselt Belgium

**Keywords:** Polycystididae, *Phonorhynchopsis*, pray, feeding, meiofauna

## Abstract

Microturbellarian flatworms comprise a diverse assemblage amongst meiofauna. These animals primarily exhibit carnivorous feeding habits, preying on various organisms, such as crustaceans, annelids and even other microturbellarians. However, details of their diet are poorly known. This study represents the first documentation of a proseriate preying upon a rhabdocoel. The proseriate was extracted from the sediment and studied alive. Within its digestive tract, structures of the reproductive systems of its prey were observed and identified as belonging to *Phonorhynchopsishaegheni*, a predatory turbellarian as well. No remains of any other organisms were detected. This finding underscores the relevance of Proseriata as top-level predators within the meiofaunal trophic web, a role that warrants further consideration beyond what has been previously acknowledged.

## Introduction

Turbellarian flatworms, along with nematodes and crustaceans, are predominant members of meiofaunal communities in terms of biodiversity and abundance (Fonseca et al. 2010, Leasi et al. 2018). The majority of these organisms act as predators or scavengers, exerting pressure on other meiofaunal members and juvenile stages of macrofauna (Bush 1966, Menn and Armonies 1999, Noreña et al. 2015). Turbellarians exhibit varied diets, encompassing diatoms, annelids, rotifers, crustaceans, nematodes and even dead animals, with no documented evidence of feeding on detritus (Graff 1904, Graff 1882, Luther 1960, Bilio 1964, Bush 1966, Straarup 1970, Martens and Schockaert 1986, Rocha et al. 1990, Ser 1991, Knaust 2021). Flatworms can specialise in feeding on specific items and develop physiological mechanisms linked to their dietary preferences. For example, some species of macrosotmids and proseriate have been found to harbour kleptocnids, cnidae acquired from cnidarian prey, which provide protection from potential predators (Karling 1966, Krohne 2018). Additionally, kleptoplasty, which consists of sequestering algal plastids that provide their host with carbon and oxygen derived from photosynthesis, has been demonstrated in rhabdocoel microturbellarians (Graff 1905, Luther 1948, Karling 1974, Rivest et al. 1999, Adam and Balzer 2001, Van Steenkiste et al. 2019). The representativies of Proseriata and Rhabdocoela orders are amongst the most diverse and prevalent microturbellarians in marine sandy habitats (Curini-Galletti 2001, Fonseca et al. 2010, Schmidt-Rhaesa 2020, Curini-Galletti et al. 2023). Proseriates are exclusively carnivorous, with records of predation on crustaceans, annelids, hydroids, ostracods and scavenging activities, even involving beached fish (Palombi 1926, Bush 1966, Bilio 1967, Murina 1981, Watzin 1983, Watzin 1985, Watzin 1986, Curini-Galletti et al. 2020). However, little is known about their interactions with other carnivorous platyhelminths or their trophic web position within the meiofaunal ecosystem. This study presents the first documentation of a proseriate preying on a carnivorous rhabdocoel.

## Material and methods

**Figure 1.**
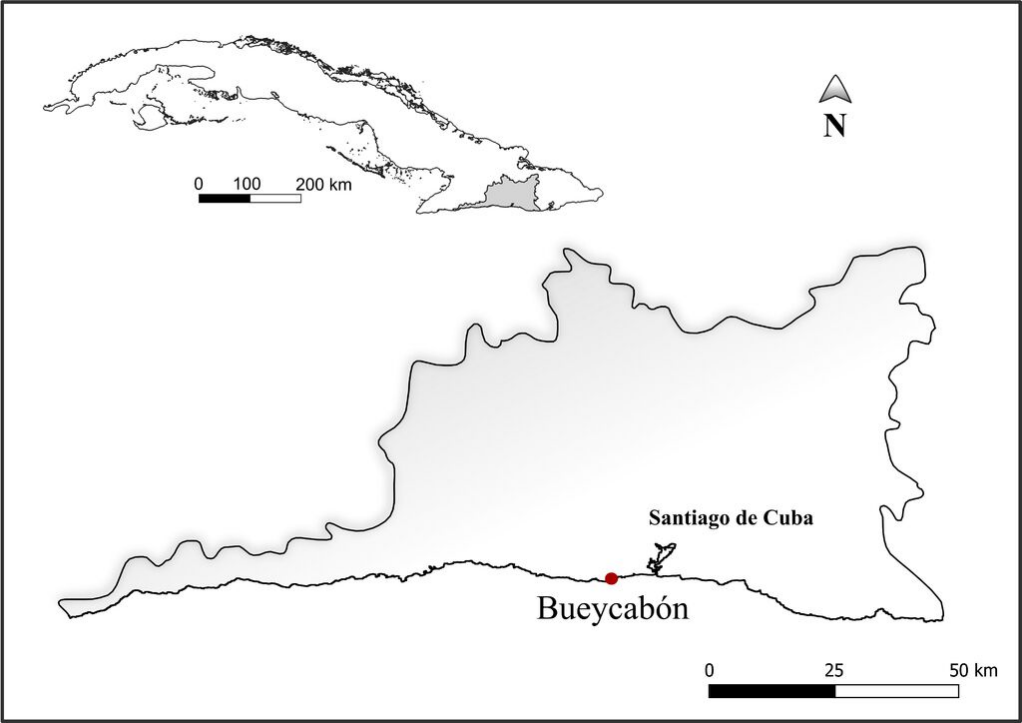
Locality of eastern Cuba where the proseriate microturbellarian was collected.

The specimen under examination was collected from the intertidal zone of Bueycabón, Santiago de Cuba, Cuba, on 18 November 2017 (Fig. 1). The sample was sourced from a depth of 0.5 min fine sand enriched with organic matter and with a salinity of 31‰. Sediment extraction was performed using the MgCl_2_ decantation method (Schockaert 1996). The accompanying fauna comprised numerous species of crustaceans, nematodes and annelids, alongside an additional specimen of the same proseriate species. No food items were observed in the additional specimen. After isolation, the specimen was studied alive and subsequently fixed in lactophenol to study its internal morphology further. The identification of the rhabdocoel remnants discovered in the proseriate gut was based on the diagnostic features outlined in existing literature (Artois and Schockaert 2001, Willems et al. 2017, Diez et al. 2018, Smith et al. 2020). The classification of the prey's genital structures follows Artois and Schockaert (2003).

## Results and Discussion

**Figure 2.**
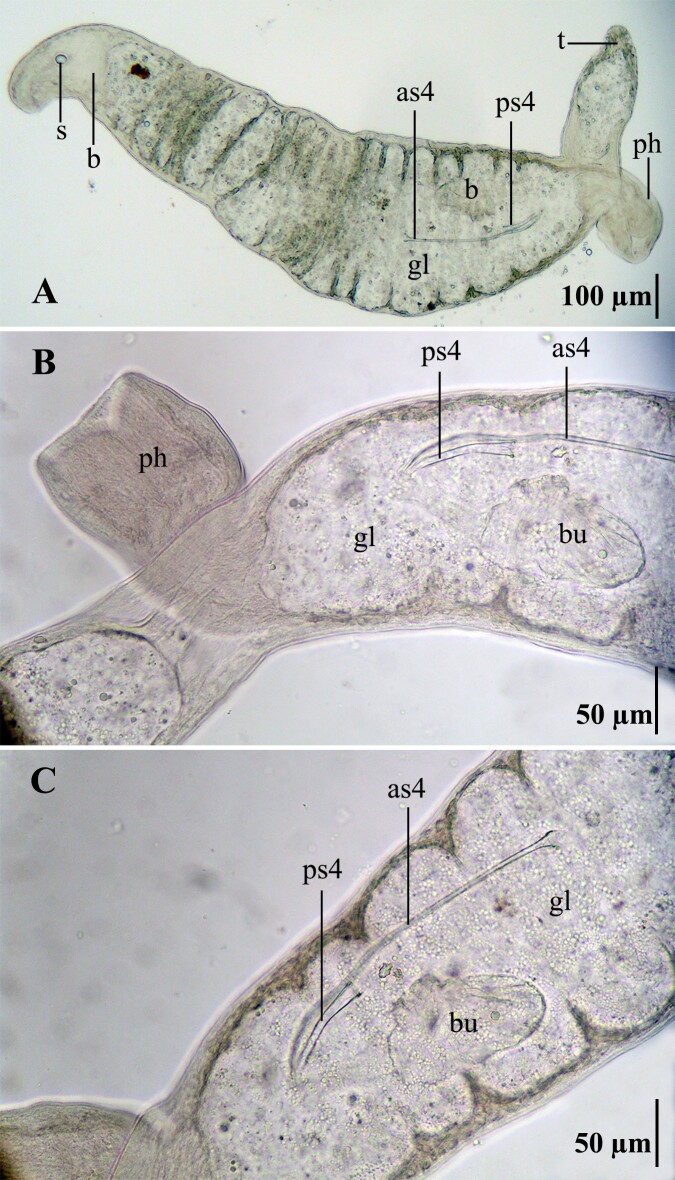
Collected specimen of Proseriata with structures of the rhabdocoel *Phonorhynchopsishaegheni* in the gut lumen. **A** complete animal body; **B, C** medial part of the animal body. Abbreviations: **as4**, accessory stylet type IV; **b**, brain; **bu**, bursa; **gl**, gut lumen; **ph**, pharynx; **ps4**, prostatic stylet type IV; **s**, statocyst; **t**, tale. **as4**, **bu** and **ps4** correspond to the rhabdocoel and the other structures to the proseriate specimen.

The collected specimen was identified as a new species of Proseriata (Fig. 2), the taxonomic description of which is presently in progress by the authors of this report. Proseriates are readily recognised by the distinctive morphology of their anterior statocyst, which sets them apart from other platyhelminths (Fig. 2, Ehler (1991)), as well as by their ‘plicatus’ type pharynx (Fig. 2, Curini-Galletti (2001)). Within the proseriate's digestive tract, we observed reproductive structures belonging to a rhabdocoel (Polycystididae Graff, 1905). These structures comprise a prostate stylet type IV (66 µm), an accessory stylet type IV (279 µm) and a part of the bipartite bursa. Willems et al. (2017) defined the presence of an accessory stylet longer than the prostate stylet and a bipartite bursa as diagnostic features of *Phonorhynchopsis* Willems & Artois, 2017 (Willems et al. 2017). A detailed study of these structures allowed us to identify the species as *Phonorhynchopsishaegheni* (Artois & Schockaert, 2001) Willems & Artois, 2017. Measurements of the sclerotised structures align with the size range previously recorded for specimens of *P.haegheni* from Cuba: a 60–100 μm long prostate stylet type IV and a 250–294 μm long accessory stylet type IV (Diez et al. 2018).

Ecological studies on proseriate feeding are scarce; the most recent ones were published over three decades ago (Luther 1960, Bilio 1964, Bilio 1967, Straarup 1970, Murina 1981, Watzin 1983, Watzin 1985, Martens and Schockaert 1986, Watzin 1986). Most insights from these works are derived from gut content analyses; direct observations of live specimen predation are sporadic (Watzin 1985). It is generally assumed that the feeding behaviour in free-living flatworms is intricately tied to pharynx morphology (Bilio 1967). Turbellarians with a simplex pharynx and a 'doliiform' pharynx are constrained to ingesting whole prey (Jennings 1957, Bilio 1967). Flatworms with a pharynx of either the 'plicatus' type (such as proseriates) or 'rosulatus' type (such as rhabdocoels) can consume entire prey or insert their pharynx into larger animals, extracting their contents and also feed on dead organisms (Meixner 1938, Jennings 1957, Bilio 1967, Straarup 1970). Bilio (1967) characterised the simplex pharynx as minimally specialised, 'doliiform' as a swallowing pharynx, 'plicatus' as a sucking pharynx and 'rosulatus' as a sucking and swallowing pharynx.

Proseriates primarily feed on copepods, nematodes, oligochaetes and polychaetes, with occasional reports of predation on cnidarians (Luther 1960, Bilio 1964, Karling 1966, Bilio 1967, Straarup 1970, Murina 1981, Curini-Galletti 2001). Watzin (1983), Watzin (1985), Watzin (1986) presented detailed accounts of the predation strategy of the monocelid *Promonotuswilsoni* (Stirewalt, Kepner & Ferguson, 1940) Martens & Curini-Galletti, 1994. This species preys on a wide array of mesopsammic organisms, including annelids and crustaceans and has even been observed preying on juvenile bivalves, nematodes, small flatworms and acoels (Watzin 1985, Watzin 1986). Notably, *P.wilsoni* displays a preference for injured crustaceans, using its protrusible pharynx to extract the entire prey or its internal organs and fluids. Partial ingestion was also observed in the case of large annelid prey organisms (Watzin 1985).

Murina (1981) reported *Pseudomonocelisophiocephala* Schmidt, 1861 targeting amphipods, oligochaetes, harpacticoids and isopods. According to the estimations of Murina (1981), in laboratory conditions, an annual density of about 14,640 individuals m^-2^ of *P.ophiocephala* can consume a massive quantity of amphipods, estimated to be up to 589,000 individuals m^-2^ per year. Straarup (1970) recorded food items of four proseriate species and compared the observations with Luther (1960),Bilio (1964), Bilio (1967): *Promonotusschultzei* Meixner, 1943, *Monocelislineata* Müller, 1774, *Monocelisunipunctata* Fabricius, 1826 and *Coelogynoporaschulzii* Meixner, 1938, all of which had diatoms or oligochaetes remains in their gut. Bilio (1964) provided a brief description of the feeding behaviour of *C.schulzii* on oligochaetes: this species attaches itself lengthwise on to a targeted oligochaete. Despite the prey's vigorous movements, *C.schulzii* connects its pharynx to the oligochaete and proceeds to extract the contents until the prey is empty. In comparison, no traces of items food previously mentioned were observed in the gut of the animal recorded in the present work.

In our studied specimen, it can be inferred that the prey specimen was similar in size compared to its predator, considering the measurements of the prey's sclerotised structures (Artois and Schockaert 2001, Diez et al. 2018). A part of the bursa of *P.haegheni* was identified in the gut lumen of the proseriate. The bursa epithelium in this species is described as pseudocuticula (Artois and Schockaert 2001). The remaining body structures of the prey seem to have already undergone digestion. According to Bilio (1967), food items with sclerotised structures, such as diatom shells, bristles of oligochaetes and spicules of nematodes, persist in the gut of flatworms for longer periods than soft tissues; if the prey has been exclusively sucked out, sclerotised structures, difficult to digest, are not ingested. As such, it may be inferred that the rhabdocoel was ingested in its entirety by the proseriate, which may be facilitated by their soft, unsclerotised bodies.

Consumption of certain turbellarians by proseriates has been documented, albeit less frequently than the consumption of other metazoan groups (Luther 1960, Bilio 1967, Straarup 1970, Watzin 1985). Anecdotal reports indicate that turbellarians are being consumed as food items by several proseriate species, including *Promonotusschultzei*, *Monocelislineata* and *Monocelisfusca* Örsted, 1843 (Bilio 1967, Straarup 1970). However, these contributions do not provide any details on the particular turbellarian groups targeted or provide details about the exerted predatory behaviour. No specific pharynx modifications or other morphological adaptations indicating a specialised diet are observed in this proseriate and we may, therefore, speculate that this species is a generalist predator. In contrast, some proseriates that prey on specific organisms, such as Cnidaria, exhibit morphological modifications in their pharynx (Karling 1966).

*Phonorhynchopsishaegheni* primarily feeds on crustaceans and nematodes, as evidenced by the presence of cuticles from these organisms in its gut (personal observations). With a documented presence in Florida, The Galapagos, Curaçao, Indonesia and Cuba (Artois and Schockaert 2001, Diez et al. 2018, Diez et al. 2023, WoRMS 2023), it is evident that this species thrives in a wide range of tropical regions. In eastern Cuba specifically, it was previously recorded in the intertidal zones of the North and South coasts, inhabiting areas with salinity levels between 30–35‰ and sediments ranging from coarse to fine sand (Diez et al. 2018). Furthermore, Diez et al. (2023) identified numerous specimens of *P.haegheni* in the same locality where this study was conducted. Considering the similar habitat preferences of the proseriate detailed in this study, encounters between both species are likely to occur. An additional factor to consider is the occurrence of the zoological groups mentioned in literature as common food sources for the proseriate within the sample. This suggests that predation on the rhabdocoel is not linked to the absence of typical food sources for Proseriata. Given that *P.haegheni* is a predator (personal observations), the observed proseriate appears to occupy a top-tier position in the food web of the meiofaunal ecosystem.

Trophic relationships with the meiofauna community may be more intricate than previously understood. While this study demonstrates predation by a proseriate on a rhabdocoel, predation on Proseriata by other meiofauna members, including rhabdocoels, has also been observed (Curini-Galletti 2001). For instance, the rhabdocoel *Pseudograffillaarenicola* Meixner, 1938 (Graffillidae Graff, 1905) preys mainly on monocelids and relatively high residential turbellarian densities may be a prerequisite for this rhabdocoel to thrive (Bilio 1964). Indeed, *Monocelislineata* is commonly found co-occurring with *P.arenicola* and could make up the main prey for the latter species (Bilio 1964). From the above and our observations, it is evident that both rhabdocoels and proseriates occupy top positions in meiofaunal feeding networks and predator-prey relationships exist in both directions between these two lineages. As members of both taxa may also engage in competition for resources, the resulting dynamics are likely intricate and require further study.

## Final considerations

Proseriates' feeding tendencies suggest they influence other meiofauna populations. As a result, these animals might shape meiofaunal communities by competing with and regulating other turbellarians like rhabdocoels, with whom they share habitats and dietary preferences. This highlights the intricate and yet-to-be-fully-understood dynamics amongst predatory meiofauna taxa. Furthermore, their predation on juvenile stages of macrofauna could also potentially impact the structure of macrofaunal communities. As such, we here argue that a deeper exploration of proseriate's ecological role is vital for a holistic understanding of marine ecosystem intricacies. It is also worth mentioning that the proseriate object of this report and presently under study, could not be easily assigned to any known genera and even families of Proseriata and may be taken as explicative of how little is still known about the rich and diverse Cuban meiofauna (Diez et al. 2023).

## References

[B10848406] Adam M. C.M., Balzer I., Seckbach J. (2001). Symbiosis: Mechanisms and model systems.

[B10848419] Artois T., Schockaert E. (2001). Interstitial fauna of the Galapagos: Duplacrorhynchinae, Macrorhynchinae, Polycystidinae, Gyratricinae (Platyhelminthes: Polycystididae). Tropical Zoology.

[B10848428] Artois T., Schockaert E. (2003). Primary homology assessment in the male atrial system of the Polycystididae (Platyhelminthes: Eukalyptorhynchia. Zoologischer Anzeiger-A Journal of Comparative Zoology.

[B10848437] Bilio M. (1964). Die aquatische Bodenfauna von Salzwiesen der Nord‐und Ostsee I. Biotop und Ökologische Faunenanalyse: Turbellaria. Internationale Revue der Gesamten Hydrobiologie und Hydrographie.

[B10848446] Bilio M. (1967). Nahrungsbeziehungen der Turbellarien in Küstensalzwiesen. Helgoländer Wissenschaftliche Meeresuntersuchungen.

[B10848455] Bush L. F. (1966). Distribution of sand fauna in beaches at Miami, Florida. Bulletin of Marine Science.

[B10848464] Curini-Galletti M., Littlewood T. J., Bray R. A. (2001). Interrelationships of the Platyhelminthes.

[B10848477] Curini-Galletti M., Artois T., Di Domenico M., Fontaneto D., Jondelius U., Jörger K, Leasi F, Martínez A., Norenburg J., Sterrer W, Todaro M. A. (2020). Contribution of soft-bodied meiofaunal taxa to Italian marine biodiversity. The European Zoological Journal.

[B10848493] Curini-Galletti M., Fontaneto D., Martinez A. (2023). Diversity of Platyhelminthes
Proseriata in Western Mediterranean sandy beaches: a database of species occurrences and traits. Biogeographia-The Journal of Integrative Biogeography.

[B10848514] Diez Y. L., Sanjuan C., Reygel P., Roosen P., Artois T. (2018). First record of Polycystididae (Platyhelminthes, Kalyptorhynchia) from Cuba, with the description of a new genus and five new species, and remarks and the description of one new species from Panama. Zootaxa.

[B10848502] Diez Y. L., Sanjuan C., Bosch C., Catalá A., Monnens M., Curini-Galletti M., Artois T. (2023). Diversity of free-living flatworms (Platyhelminthes) in Cuba. Biological Journal of the Linnean Society.

[B10848974] Ehler U. (1991). Comparative morphology of statocysts in the Plathelminthes and the Xenoturbellida.

[B10848650] Fonseca V. G., Carvalho G. R., Sung W., Johnson H. F., Power D. M., Neill S. P., Packer M., Blaxter M. L., Lambshead P. J.D., Thomas W. K., Creer S. (2010). Second-generation environmental sequencing unmasks marine metazoan biodiversity. Nature Communications.

[B10848665] Graff L. (1882). Monographie der turbellarien.

[B10848673] Graff L. (1904). Acoela und Rhabdocoelida.

[B10848681] Graff L. (1905). Marine Turbellarien Orotavas und der Küsten Europas. Ergebnisse einiger, mit Unterstützung der kais. Akademie der Wissenschaften in Wien (aus dem Legate Wedl) in den Jahren 1902 und 1903 unternommen Studienreise. II. Rhabdocoela. Zeitschrift für Wissenschaftliche Zoologie.

[B10848690] Jennings J. (1957). Studies on feeding, digestion, and food storage in free-living flatworms (Platyhelminthes: Turbellaria). The Biological Bulletin.

[B10848699] Karling T. G. (1966). On nematocysts and similar structures in turbellarians. Annales Zoologici Fennici.

[B10848708] Karling T. G. (1974). Turbellarian fauna of the Baltic Proper: identification, ecology and biogeography. Fauna Fennica.

[B10848717] Knaust D. (2021). Foraging flatworms and roundworms caught in the act: examples from a Middle Triassic mud flat in Germany. Lethaia.

[B11390468] Krohne G. (2018). Organelle survival in a foreign organism: Hydra nematocysts in the flatworm *Microstomumlineare*. *European Journal of Cell Biology*.

[B10848726] Leasi F., Sevigny J. L., Laflamme E. M., Artois T., Curini-Galletti M., Navarrete A., Di Domenico M., Goetz F., Hall J. A., Hochberg R., Jörger K., Jondelius U., Todaro A., Wirshing H., Norenburg J., Thomas W. (2018). Biodiversity estimates and ecological interpretations of meiofaunal communities are biased by the taxonomic approach. Communications Biology.

[B10848741] Luther A. (1948). Untersuchungen an Rhabdocoelen Turbellarien-VII-Uber einige marine Dalyellioida.-VII-Beitrage zur Kenntnis der Typhloplanoida. Acta Zoologica Fennica.

[B10848750] Luther A. (1960). Die Turbellarien Ostfennoskandiens. I. Acoela, Catenulida, Macrostomida, Lecithoepitheliata, Prolecithophora, und Proseriata. Fauna Fennica.

[B10848759] Martens P. M., Schockaert E. R. (1986). The importance of turbellarians in the marine meiobenthos: a review. Hydrobiologia.

[B10848768] Meixner J. (1938). Turbellaria (Strudelwürmer) in Tierwelt der Nord‐und Ostsee.

[B10848776] Menn I., Armonies W. (1999). Predatory *Promesostoma* species (Plathelminthes, Rhabdocoela) in the Wadden Sea. Journal of Sea Research.

[B10848785] Murina G. (1981). Notes on the biology of some psammophile Turbellaria of the Black Sea. Hydrobiologia.

[B10848794] Noreña C., Damborenea C., Brusa F., Thorp JH., Rogers CD. (2015). Thorp and Covich's freshwater invertebrates.

[B10848807] Palombi A. (1926). *Digenobothriuminerme* nov. gen. nov. sp. (Crossocoela). Considerazioni sistematiche sull'ordine degli Alloeocoela. Archivio Zoologico Italiano.

[B10848816] Rivest B. R., Coyer J., Tyler S. (1999). The first known invasion of a free-living marine flatworm. Biological Invasions.

[B10848825] Rocha O., Matsumura-Tundisi T., Galizia Tundisi J., Padovesi Fonseca C. (1990). Predation on and by pelagic Turbellaria in some lakes in Brazil. Hydrobiologia.

[B10848834] Schmidt-Rhaesa A. (2020). Guide to the identification of marine meiofauna.

[B10848843] Schockaert E., Hall G. (1996). Methods for the examination of organismal diversity in soils and sediments.

[B10848856] Ser M. (1991). Meiofauna of an experimental soft bottom ecosystem-effects of macrofauna and cadmium exposure. Marine Ecology Progress Series.

[B10848865] Smith J. P., Van Steenkiste N., Artois T., Schmidt-Rhaesa A. (2020). Guide to the identification of marine meiofauna.

[B10848878] Straarup B. J. (1970). On the ecology of turbellarians in a sheltered brackish shallow-water bay. Ophelia.

[B10848887] Van Steenkiste N. W., Stephenson I., Herranz M., Husnik F., Keeling P. J., Leander B. S. (2019). A new case of kleptoplasty in animals: marine flatworms steal functional plastids from diatoms. Science Advances.

[B10848898] Watzin M. C. (1983). The effects of meiofauna on settling macrofauna: meiofauna may structure macrofaunal communities. Oecologia.

[B10848907] Watzin M. C. (1985). Interactions among temporary and permanent meiofauna: observations on the feeding and behavior of selected taxa. The Biological Bulletin.

[B10848916] Watzin M. C. (1986). Larval settlement into marine soft-sediment systems: interactions with the meiofauna. Journal of Experimental Marine Biology and Ecology.

[B10848925] Willems W. R., Reygel P., Van Steenkiste N., Tessens B., Artois T. J. (2017). Kalyptorhynchia (Platyhelminthes: Rhabdocoela) from KwaZulu-Natal (South Africa), with the description of six new species. Zootaxa.

[B10848935] WoRMS Phonorhynchopsis haegheni. https://www.marinespecies.org/aphia.php?p=taxdetails&id=1063074.

